# The effect of ranolazine on maintaining sinus rhythm in patients with resistant atrial fibrillation

**Published:** 2008-08-01

**Authors:** David K Murdock, Naomi Overton, Mary Kersten, Jeff Kaliebe, Fausto Devecchi

**Affiliations:** Cardiovascular Associates of Northern Wisconsin, S.C., CaRE Foundation, Inc., USA

**Keywords:** atrial fibrillation, ranolazine, cardiac arrhythmias

## Abstract

**Background:**

Atrial fibrillation (AF) may arise out of anomalous impulse activity at atrial venous junctions. Triggered activity may be a source of abnormal impulse activity. Ranolazine is an anti-anginal agent, which inhibits normal and abnormal late Na+ channel current in the ventricle and peak Na+ channel current in the atrium. This produces an energy sparing effect and stabilizes cardiac membranes. Ranolazine is a potent inhibitor of triggered activity. The purpose of this report is to describe our initial experience with ranolazine used in patients with resistant AF.

**Methods:**

Seven patients (4 males, 3 females, 67 ± 9 years) who developed recurrent AF within hours to a few days of restoring sinus rhythm despite AF ablation and /or failing one or more anti-arrhythmic agents were started on ranolazine (500-1000 mg/twice/day) after stopping all other anti-arrhythmic therapy. All but one patient had some form of associated structural heart disease.

**Results:**

Two patients received no apparent benefit from ranolazine developing recurrent AF within 2 days.  All other patients derived significant benefit. Four patients have experienced no recurrent AF. The other patient relapsed at 3 months and again at 6 months. The mean time in sinus rhythm to date, or to the first relapse, for the five responders was 27 ± 11 weeks.  No clinically evident pro-arrhythmic episodes occurred.

**Conclusion:**

Ranolazine was helpful in maintaining sinus rhythm in the majority of patients in which more established measures had failed. A controlled prospective trial is warranted to further investigate the efficacy of ranolazine in AF.

## Introduction

In 1998, Haissaguerre et al demonstrated atrial fibrillation (AF) may originate and be perpetuated from ectopic activity originating at the junction of the left atrium and pulmonary veins [1]. Subsequently, similar evidence has suggested that ectopic activity can also originate at the atrial junction of other thoracic veins; the vena cava and coronary sinus [2,3]. This localized source of AF forms the current basis for attempts to ablate AF by either surgical or catheter based techniques [1-3]. The electrophysiologic mechanisms responsible for the abnormal impulse activity have been the source of several investigations [1-8]. Recently it has been determined that triggered activity may be particularly important [4-8].

Ranolazine is an anti-anginal agent, which inhibits normal and abnormal late Na^+^ channel current in the ventricle and peak Na+ channel current in the atrium [9-11]. By this inhibition, it affects intracellular calcium handling producing an energy sparing effect [9]. Ranolazine has also been shown to be a potent inhibitor of after depolarizations produced by a number of mechanisms [10,12,13]. As such, it could prove to be particularly useful in the treatment of AF. Indeed, in the holter monitor data from the MERLIN trial, ranolazine was associated with a reduction in a number or several arrhythmias, including new episodes of AF [14,15]. In this case series we describe our initial experience using ranolazine as the sole anti-arrhythmic agent in patients in whom standard measures to control the AF had failed. This was a retrospective analyis of our results using ranolazine for atrial fibrillation, our institutional review board does not require approval for chart review.

## Methods

Seven patients were started on ranolazine for the specific purpose of attempting to control AF after at least one anti-arrhythmic agent had failed. Each patient had been tried on at least one standard anti-arrhythmic agent (dofetilide, sotalol, propafenone, flecainide, or amiodarone) at doses felt to be the maximum tolerated or clinically safe. Two patients had also undergone catheter-based ablation. Despite these measures, each of these patients had developed recurrent AF within hours to 3 days of restoring sinus rhythm either spontaneously or by electrical cardioversion. Six of 7 patients were highly aware of their AF with noticeable palpitations. One patient did not experience palpitations and was unaware of his AF; presenting instead with congestive heart failure and severe left ventricular dysfunction. The AF was classified as "persistent" if it failed to self convert and required electrical cardioversion to restore sinus rhythm. AF was classified as paroxysmal if the AF alternated with sinus rhythm via spontaneous relapses and self-conversion.

The age, sex, left ventricular ejection fraction (%), left atrial diameter (mm) and volume index (ml/m^²^) by echocardiography, number of anti-arrhythmic agents tried prior to ranolazine (not including beta blockers or calcium channel blockers), and associated structural heart condition and other health conditions were determined for each patient. The nature of the AF problem (paroxysmal or persistent) was also determined for each patient.

Each patient was advised that this was an "off label" use of ranolazine and that the benefits and risks were unknown. Ranolazine was started only after stopping other anti-arrhythmic therapy. Ranolazine was started at 500 mg twice a day and if tolerated generally increased within 2 to 4 days to 1000 mg twice a day in patients with a weight exceeding 70 kg. If the patient was on amiodarone immediately prior to ranolazine, amiodarone was discontinued at least 4 weeks before ranolazine was begun. All other anti-arrhythmic agents were discontinued at least 3 half lifes (if in hospital with ECG monitoring, 4 patients) or 5 half lifes if stated in the unmonitored or outpatient setting (3 patients). Patients were not hospitalized for ranolazine initiation. Those in the hospital at the time it was started had been admitted for standard anti-arrhythmic therapy, but failed therapy within a day of cardioversion. Four patients were in AF when ranolazine was started and were cardioverted to sinus rhythm within a few days. Three patients had incessant paroxysmal AF and were in sinus rhythm when started on ranolazine. The duration in weeks for all patients who remained in sinus rhythm for at least 1 week after restoring sinus rhythm was determined and the mean  ± 1 standard deviation for the group calculated. The specific details for anybody relapsing into AF was noted and reported.

## Results

Table 1 describes the clinical characteristics of the patients in which ranolazine was used to treat AF. Note the wide spectrum of associated medical conditions and structural heart disease. Six of the 7 patients had left atrial enlargement as measured by an elevated left atrial volume index exceeding 26 ml/m^²^.

Table 2 details the clinical effect of ranolazine on AF as well as the dose employed. Ranolazine was effective in maintaining sinus rhythm in 5 patients. Four of these five have had no recurrent AF. One of the 5 patients (#1) did develop recurrent AF. This patient had a 12-year history of lone AF (normal left atrial size initially). AF was initially infrequent and controlled with beta-blockers and occasional cardioversion. As left atrial size increased over time, the AF became increasingly problematic. Amiodarone, dofetilide, sotalol, and flecainide had each failed. The patient under went catheter-based ablation at an outside institution. After a brief period of benefit, recurrent AF led to 3 repeat cardioversions whereby sinus rhythm would last only 1 to 3 days. Another ablation was contemplated. Instead ranolazine was started as an outpatient and the patient electrically cardioverted 3 days later. Normal sinus rhythm persisted for 12 weeks before recurrent AF occurred. After electrical conversion on ranolazine, sinus rhythm was again maintained for another 12 weeks before a second relapse occurred. Following this second relapse, ranolazine was abandoned as a therapeutic option and the patient now remains in chronic AF with possible plans for a second attempt at radio frequency ablation.

Two patients had no apparent benefit from ranolazine. Both patients had incessant paroxysmal AF with several episodes of AF each day. Both failed numerous anti-arrhythmic agents without any apparent clinical benefit. Both continued to have incessant paroxysmal AF despite ranolazine at 2000 mg/day.

All other patients remain in sinus rhythm without relapse despite having had very challenging AF. The mean time in sinus rhythm to date, or to the first relapse, for the five responders was 27 ± 11 weeks.   No clinically evident pro-arrhythmic events were noted in any patient. Nobody discontinued ranolazine because of side effects.

## Discussion

Maintaining sinus rhythm in patients with AF can be particularly challenging. Despite anti-arrhythmic therapy, AF continued to be highly problematic in each of our patients. Each patient with paroxysmal AF had daily episodes of AF.  Each patient with persistent AF had undergone numerous cardioversions with rapid relapse to AF.  Since each patient had highly symptomatic AF (6 patients) or experienced a severe deterioration of left ventricular function as a consequence of their AF (1 patient), we pursued an aggressive approach to AF in these patients.

We found ranolazine to be effective in suppressing AF in the majority of patients with persistent AF stemming from a variety of causes (Table 2). Most patients with persistent AF remain in sinus rhythm as of this writing (Table 2). Prior to ranolazine each of these patients had relapsed to AF within 3 days of last cardioversion. However, like all pharmacological agents, ranolazine did not prevent all AF. It appeared to have little effect in 2 of the 3 patients with frequent paroxysmal AF.

The possible utility of ranolazine to treat clinical arrhythmias is only now beginning to emerge. In 6560 patients surviving an acute coronary syndrome, the MERLIN investigators noted that ranolazine was associated with a significant reduction in a variety of asymptomatic atrial and ventricular arrhythmias as recorded on prolonged holter monitoring [14,15]. Importantly, in extensive clinical studies to date, the pro-arrhythmic potential of ranolazine appears to be very low [14,16]. None of the patients in our series had a symptomatic pro-arrhythmic event.

The mechanism for the anti-arrhythmic effects observed with ranolazine is likely complex. Ranolazine slightly prolongs the action potential duration by inhibiting the slow sodium current and the slow component of the delayed rectifying potassium current [12].  In atrial tissue, the inhibition of the peak sodium current prolongs repolarization and induces post repolarization refractoriness [11].  This effect could potentially block re-entrant pathways. However, it is its effect on triggered activity, which appear most powerful. In tissue bath preparations, ranolazine reduces the repetitive extra systoles produced by triggered activity from a number of different causes [10,12,13]. Ranolazine affectively prevents or attenuates AF in canine models of this arrhythmia [11].

## Limitations

The small number of patients in our report makes it essential that these observations be confirmed. Additionally, this was a real life experience with ranolazine. Like all real life clinical decision making regarding anti-arrhythmic therapy, we gauged the effectiveness of ranolazine based on the past history of very resistant AF and the significant change in that pattern after institution of ranolazine. Finally, we used ranolazine as a last resort measure only. It is possible that these criteria artificially selected a group of patients more likely to respond to ranolazine. The use of ranolazine for less resistant AF remains uninvestigated.

## In summary

Our investigation confirms the anti-arrhythmic properties of ranolazine and is the first report on the specific use of ranolazine to treat symptomatic AF.  We found ranolazine to be effective in suppressing AF in the majority of patients with resistant AF stemming from a variety of causes. Given the lack of known pro-arrhythmic potential and end organ toxicity, as well as the ability to initiate therapy in an outpatient setting, the potential implications of the present observations are obvious. If the effectiveness of ranolazine is confirmed in larger controlled trial, this agent could emerge as the drug of first choice for AF rather than the manner we employed it. The small number of patients demands us to be cautious before extrapolating to the larger clinical setting and highlights the need for larger controlled studies to investigate the possible utility of ranolazine to treat this difficult and pervasive problem.

## Figures and Tables

**Table 1:**
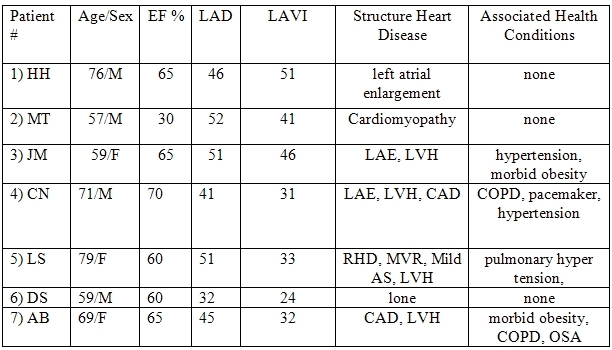
Clinical Characteristics of AF patients

AF: atrial fibrillation, CAD: coronary artery disease, COPD: chronic obstructive pulmonary disease, EF: ejection fraction, LAD: left atrial diameter (mm), LAE: left atrial enlargement, LAVI: left atrial volume index (ml/m^²^), LVH: left ventricular hypertrophy, OSA: obstructive sleep apnea, RHD: rheumatic heart disease.

**Table 2:**
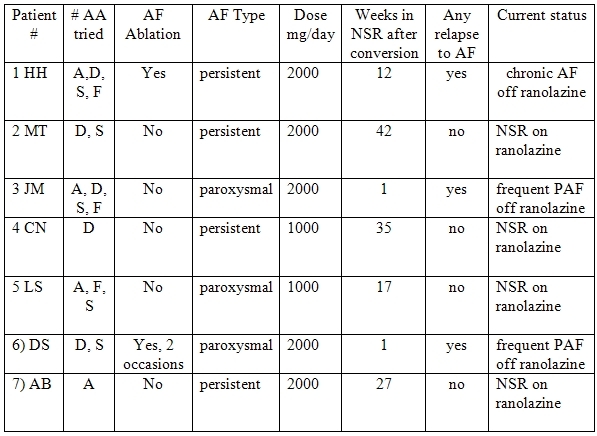
AF Characteristics and Ranolazine effects

A: amiodarone, D: dofetilide, S: sotalol, F: flecainide, NSR: normal sinus rhythm, PAF: paroxysmal atrial fibrillation
